# Dog Movie Stars and Dog Breed Popularity: A Case Study in Media Influence on Choice

**DOI:** 10.1371/journal.pone.0106565

**Published:** 2014-09-10

**Authors:** Stefano Ghirlanda, Alberto Acerbi, Harold Herzog

**Affiliations:** 1 Department of Psychology, Brooklyn College, Brooklyn, New York, United States of America; 2 Centre for the Study of Cultural Evolution, Stockholm University, Stockholm, Sweden; 3 Department of Archaeology and Anthropology, University of Bristol, Bristol, United Kingdom; 4 Department of Psychology, Western Carolina University, Cullowhee, North Carolina, United States of America; Durham University, United Kingdom

## Abstract

Fashions and fads are important phenomena that influence many individual choices. They are ubiquitous in human societies, and have recently been used as a source of data to test models of cultural dynamics. Although a few statistical regularities have been observed in fashion cycles, their empirical characterization is still incomplete. Here we consider the impact of mass media on popular culture, showing that the release of movies featuring dogs is often associated with an increase in the popularity of featured breeds, for up to 10 years after movie release. We also find that a movie's impact on breed popularity correlates with the estimated number of viewers during the movie's opening weekend—a proxy of the movie's reach among the general public. Movies' influence on breed popularity was strongest in the early 20^th^ century, and has declined since. We reach these conclusions through a new, widely applicable method to measure the cultural impact of events, capable of disentangling the event's effect from ongoing cultural trends.

## Introduction

Fashions and fads are ubiquitous in modern societies [1], [2], as well as in “traditional” societies [3] and in past societies [4], and have been studied in disciplines as diverse as philosophy, sociology, anthropology, and economics [5]–[10]. Recently, fashions have received renewed attention as a source of data to test models of cultural dynamics [11]–[14]. In this context, fashions and fads are defined intuitively as cultural traits whose popularity undergoes striking fluctuations (often short-term) that do not have any obvious cause, and therefore appear whimsical or erratic. Some statistical regularities have nevertheless been found.

Bentley and coworkers showed that, in many cultural domains, relatively few traits are common while the vast majority are very rare (trait frequency follows log-normal or power law distributions, see [12], [13], [15]). They also showed that the hypothesis that individuals copy each other at random is sufficient to explain this pattern. Other findings, however, challenge the idea that chance dominates cultural dynamics. Popularity trends may have a consistent direction for many years [16], while random copying generally predicts no correlation between years. Furthermore, rates of increase in popularity appear correlated with rates of decrease: what becomes popular rapidly is also rapidly forgotten [14], [17]. Berger and coworkers have also showed that the popularity of a first name is influenced by the popularity of phonetically similar names [18]. Several models have been developed to accommodate these findings [14], [16], [17].

This paper continues the search for quantitative data in order to better characterize cultural dynamics. In particular, we ask whether it is possible to detect the effect of a specific class of events on fashion dynamics. Within this broader context, we have investigated whether the release of movies featuring dogs is associated with changes in the popularity of featured breeds. This choice was motivated by high interest of the general public in both dogs and movies, and by the availability of good quality data. We show that, indeed, movies have had a significant impact on dog breed popularity in the U.S.A., sometimes influencing sales of featured breeds for a decade or more, but also that their effect has been declining over time. Our results show that, while fashions may appear erratic, it may be possible, at least sometimes, to identify specific underlying causes.

## Methods

### Data sources

The American Kennel Club (AKC) maintains the world's largest dog registry and provided us with the number of registrations for each recognized breed between 1926 and 2005, totaling over 65 million registered dogs (see [19], [20] for details). To identify movies featuring dogs, we used the following Internet resources: http://www.caninest.com/dog-movies, http://en.wikipedia.org/wiki/List_of_fictional_dogs#Dogs_in_film, and http://www.disneymovieslist.com/best/top-dog-movies.asp, retrieved between August and September, 2012. The results of our search and successive data selection are summarized below. The data are publicly available [21].

We located 87 movies featuring dogs, of which 81 had been released in the U.S.A. between 1927 and 2004 (the years for which we can calculate at least one-year trend changes). Of these, 63 featured a breed for which data is available in the AKC database. We excluded four movies because the dog was not a main character: *Thin man* (Metro-Goldwyn-Mayer, 1934), *The Swiss family Robinson*, (Walt Disney, 1960), *The nightmare before Christmas* (Touchstone Pictures, 1993), and *Meet the Fockers* (TriBeCa Productions, 2004). Dogs that we considered “main characters” are typically mentioned in the movie title or prominently featured in movie synopses. We excluded the movie *Cujo* (Taft Entertainment, 1983) because the dog is a negative character. Of the remaining 59 movies, some featuring the same breed were released only a few years apart. For example, there are seven movies of the *Lassie* series released between 1943 and 1951, all featuring a collie as the main character. It would be statistically unsound to include all of these movies in our analysis because the impact of different movies on the popularity of collies would then be estimated based partly on the same data. To safeguard the independence of data points entering statistical analysis, we retained movies featuring the same breed only if they were released more than 20 years apart. We could thus compute breed popularity trends for up to 10 years before and after movie release. When we found movies featuring the same breed, we retained the earliest one for analysis, and moved forward in time to include the first movie released more than 20 years later, and so on until all movies were either included or excluded from analysis. In the case of collies, for example, we retained *Lassie* movies released in 1943 and 1978, excluding seven movies released in 1945–1963 and one movie released in 1994. This step of data selection resulted in the retention of 30 movies. Of these we had to exclude *The Plague Dogs* (Embassy Pictures, 1982) because the featured breed (the smooth fox terrier) was not recognized by the AKC in 1982. The final data set included thus 29 movies. One movie featured four breeds, and four movies featured two, resulting in a total of 36 data points.

By excluding some movies for the purpose of statistical analysis we do not mean to imply that these movies have had not effect on breed popularity. For example, the rise in the popularity of collies observed after the release of the first *Lassie* movie in 1943 may have been partly caused by movies with the same character released in the next few years. In the following, we leave it understood that the effects that, nominally, we attribute to one movie may have been caused by several movies.

We estimated the number of viewers for each movie by dividing the movie's U.S.A. earnings by the average movie ticket price at the time of movie release. These data were obtained from Box Office Mojo (http://boxofficemojo.com, preferred) or the English language Wikipedia entry of the movie (http://en.wikipedia.org). Ticket prices were missing for some years, and were linearly interpolated based on adjacent years. We found total earnings for 23 of the 29 movies retained for analysis. We also found earnings during the opening-weekend for 16 movies.

### Estimate of movie effect

**Figure 1:**
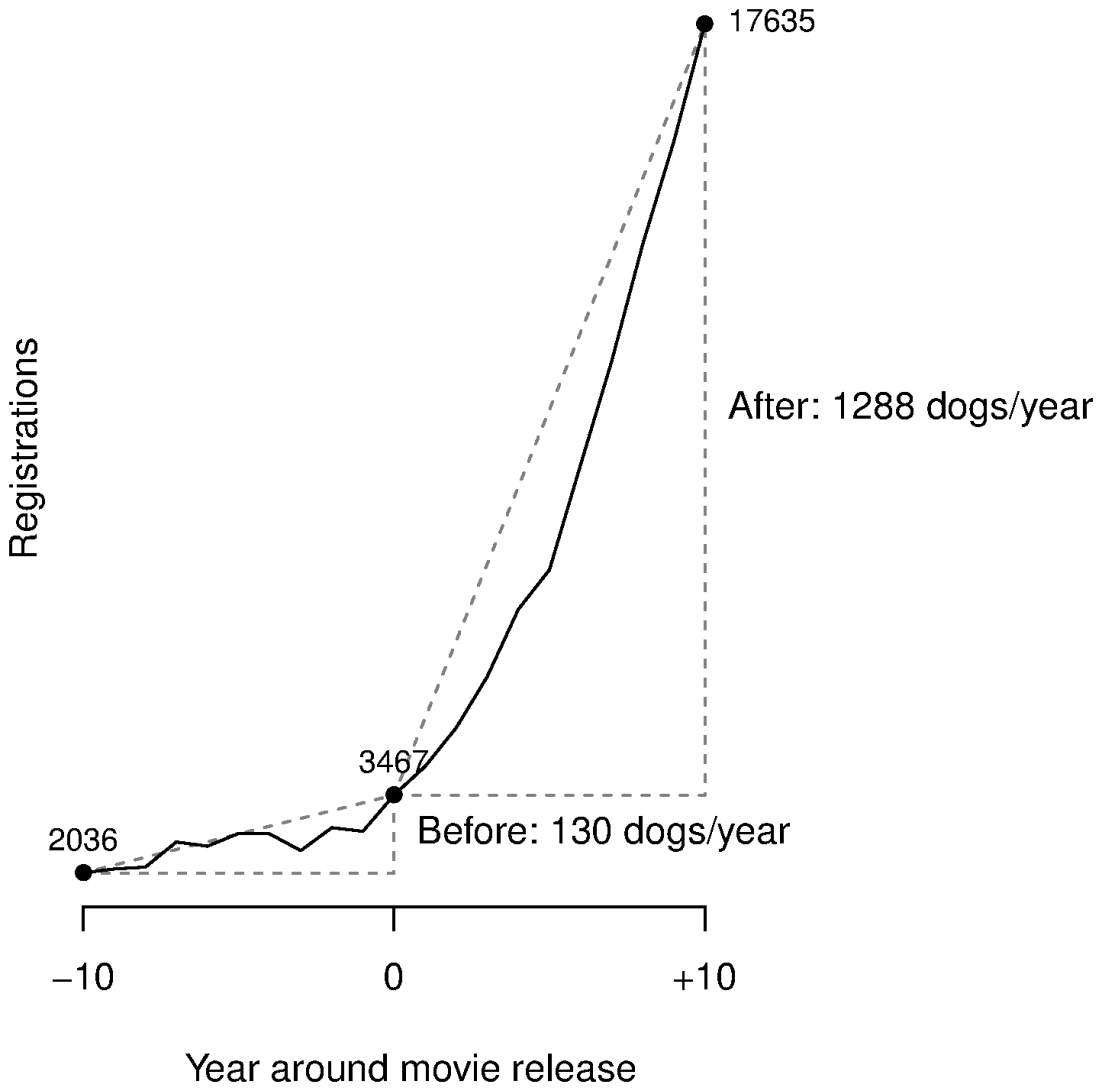
Estimation of a movie's effect on breed popularity. American Kennel Club data show that registrations of Labrador retrievers increased at an average rate of 452 dogs/year in the 10 years preceding the release of *The incredible journey* (Walt Disney, 1963), and at an average rate of 2223 dogs/year in the 10 following years. Over the 21 years surrounding movie release registrations occurred at an average of 13483 dogs/year. Thus equation (1) yields an estimated 10-year effect of the movie of 
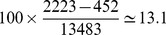
. Note that registrations were already increasing before movie release, but increased faster afterward.

The effect of a movie on breed popularity cannot be estimated simply by looking for an increase in breed registrations after movie release. Such an increase, in fact, could be part of a trend in breed popularity that had started before movie release. Indeed, it is possible that a breed is chosen for a movie precisely because it is becoming popular. Thus we study the effect of movies by investigating changes in registration *trends* rather than in registrations *per se*. We have constructed an index of trend change such that a value of 100 means that after movie release *per capita* registrations increased 100% over what was expected based on the pre-release trend (Fig. 1).

Formally, we define the 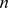-year trend change associated with a movie, 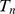, as the percentage change in yearly breed registrations between the 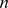 years preceding movie release and the 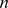 years following it, divided by the average number of registrations per year over the considered period:
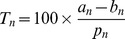
(1)where 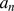 is the average change in registrations in years 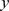 to 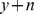 (after movie release), 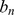 is the average change in registrations in years 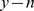 to 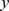 (before movie release), and 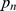 is the average number of registrations per year between years 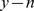 and 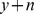.

Using this method, we investigated trends over periods of 1, 2, 5, and 10 years. We report estimated 1-year trends for completeness, but we note that they may be less reliable than estimates of longer trends because they are more influenced by such factors as the time of movie release (e.g., Christmas vs. Easter), delays in dog registrations by owners, and delays in registration processing by the AKC. A graph of all 10-year trends is publicly available [22]. All statistical analyses were performed with R, version 3.0.0 [23].

## Results

**Figure 2:**
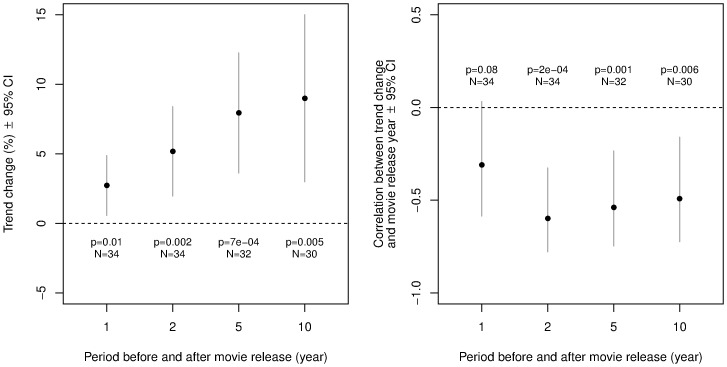
Left: Average trend changes in breed registration around the year of movie release, for trend changes measured over four different time spans. Statistical significance and confidence intervals are based on two-tailed one-sample 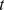-tests. Sample sizes vary because, given availability of breed registration data for 1926–2005, we could perform 1-, 2-, 5- and 10-year trend analysis for movies released, respectively, in 1927–2004, 1928–2003, 1931–2000, and 1936–1995. Right: Correlation between trend change and movie release year. Negative correlations mean that later movies tend to be associated with smaller trend changes.

**Figure 3:**
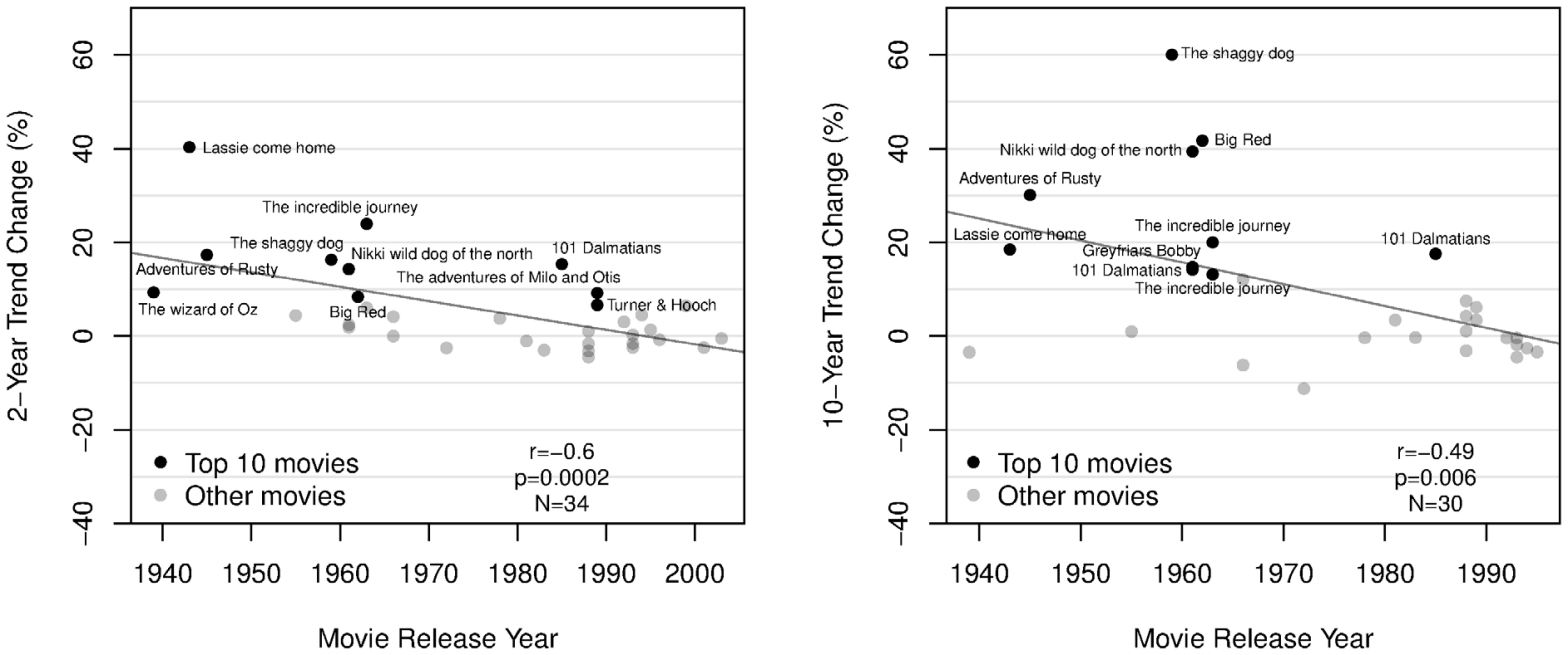
Changes in breed registration trends associated with movie release, calculated according to equation (1). Left: 2-year changes. Right: 10-year changes. The 10 movies associated with the greatest trend changes are highlighted. Statistical information at the bottom of each panel refers to linear fits to the data (gray lines). The movie *Snow dogs* (Walt Disney, 2002) features Siberian huskies and a border collie, and is associated with large 2-year trend changes for both breeds (rightmost labeled points in the left panel; collies are the top point). Similarly, the movie *The incredible journey* (Walt Disney, 1963) featured both golden retrievers (labeled) and bulldogs. Several releases of, or sequels to *101 Dalmatians* appear in both panels.

The average trend change associated with movie release is significantly greater than zero over 1-, 2-, 5-, and 10-year periods (Fig. 2, left). In addition, we find a strong negative correlation between trend change and movie release year for 2-, 5-, and 10-year trends (Fig. 2, right; Fig. 3). Thus earlier movies are associated with generally larger trend changes than later movies. Two possible reasons for the decreasing impact of movies are increased competition from other media, such as home video, as well as increased competition among movies. Movies featuring dogs, for example, were released at a rate of less than one per year until about 1940, but at a rate of more than 7 per year by 2005 (as estimated by a linear fit to the data, binned in 5-year periods; Pearson's correlation between number of movies and year is 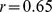, 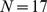, 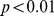, two-tailed).

Movie-associated trend changes correlate significantly with the estimated number of viewers during the movie's opening weekend. We constructed linear models with movie effect as dependent variable, and number of opening weekend viewers (log-transformed) and release year as independent variables. We found a significant main effect of year for 5-, and 10-year trends (5 years: 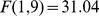, 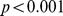; 10 years: 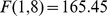, 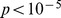), and a non-significant effect for 1- and 2-year trends (1 year: 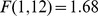, 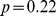; 2 years: 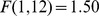, 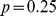). We also found a significant main effect of number of opening-weekend viewers for 1-, 5-, and 10-year trends (1 year: 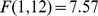, 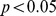; 5 years: 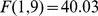, 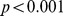; 10 years: 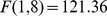, 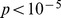), and a non-significant effect for 2-year trends (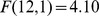, 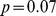). There was no significant interaction between release year and number of viewers (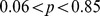). (Log-transformed number of viewers does not correlate with release year: Pearson's 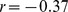, 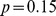, or Spearman's 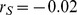, 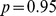; 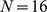.) These results suggest that the number of viewers during a movie's opening weekend is a good proxy of the movie's future impact on popular culture. In a similar set of linear models, we found no significant effect of the *total* number of viewers on 1-, 2-, 5-, and 10-year trends (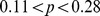). A possible reason for this discrepancy is that estimates of total viewers cover extended periods, thus are expected to correlate less with trend changes around the time of movie release.

Overall, these data suggest that viewing a movie may cause a long-lasting preference for a breed that can be expressed years later, e.g., when the time comes to buy a new dog. Indeed, trend changes appear to increase when measured over longer periods (Fig. 2, left). For example, 14 out of cases for which 10-year trends could be calculated, are associated with stronger 10-year than 2-year trend changes.

The popularity of cultural traits is sometimes observed to undergo a nonlinear increase in which initially slow growth is replaced by faster growth [24]. Our method only compensates for linear trends, and thus would overestimate the impact of movies that, by chance, are released at the time of a transition between slower and faster growth that would have occurred anyway, independently of movie release (we are indebted to the reviewers for this observation). This potential confound does not appear to affect our data. In fact, we find that in about a third of cases breed popularity was *decreasing* at the time of movie release (35, 32, 34, and 33% of cases for 1-, 2-, 5, and 10-year trends, respectively). In these cases, differences in pre- and post-release popularity trends cannot derive from an ongoing transition between slower and faster growth. Additionally, we find that whether the pre-release trend is negative or positive makes no difference for estimated movie impact (two-tailed Wilcoxon tests, 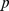 values for 1-, 2-, 5-, and 10-year trends are, respectively: 0.84, 0.70, 0.20, 0.42). Thus movies released at times of decreasing breed popularity appear as effective in boosting popularity as movies released at times of increasing popularity.

Our last point concerns feedbacks in fashion dynamics and the validity of our method to investigate such dynamics. We mentioned in Methods that a breed might have been chosen for a movie because it was perceived as “trendy” by movie producers. Indeed, we find that 10-year pre-release trends are, on average, positive in our sample (mean of 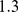
%, 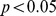, 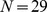, one-sample two-tailed 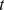-test; pre-release trend measured as 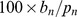, see equation 1) and that pre-release trends for the top 10 movies (Fig. 3, right) are even larger (mean of 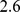
%, 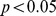, 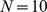, one-sample two-tailed 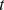-test). These data suggest that movies featuring dogs tend to use breeds whose popularity had been increasing for some time. Thus there might be a positive feedback loop whereby rising popularity can increase the chances that a breed will appear in movies, which can increase popularity further. These results hold for 10-year trends, but not for trends over shorter periods. This is expected from the fact that it takes time to notice a trend (either by casual observation or by market research), and that there is a delay of several years between the decision to use a breed and movie release.

## Discussion

While movies have been previously found capable of influencing individual behavior, for example cigarette smoking [25]–[27], our study is the first to assess the impact of movies over many decades, and the first to study a behavior—choice of dog breed—that is subject to the erratic fluctuations typical of fashions and fads [19], [28]. Our results confirm quantitatively the common belief that movies can have a lasting impact on popular culture. In the case of dog breed popularity, the impact of movies has been large. For example, the top 10 movies highlighted in Fig. 3, right, are associated with changes in registration trends such that over 800,000 more dogs were registered in the 10 years after movie release than would have been expected from pre-release trends. These results complement our recent finding that breed popularity appears unrelated to breed temperament and health [29], lending support to the idea that important aspects of people's life (in this case, their favorite pets) can be strongly influenced by fashions and fads [30].

We are aware of few studies attempting to quantify the influence of specific events on popular culture. Berger and coworkers found that book sales in the U.S. are influenced (both positively and negatively) by reviews in the *New York Times*, and that the names used for hurricanes, as well as similar names, increase in popularity among first names [18], [31]. Together with ours, these studies show that influences on popular culture can be detected given enough data. While we cannot be sure that a single movie, newspaper review, or hurricane can influence culture, pooling data for many similar events can reveal consistent trends. In the quest to understand what influences popular culture, negative results can also be informative. We previously found, for example, that breeds that win the Westminster Kennel Club Dog Show do not, on average, increase in popularity [20], suggesting that reaching a small specialized audience may not be as effective as reaching the general public.

Lastly, we recall that we have focused on popularity trends rather than on popularity itself, in order to avoid attributing to movies trends that were already ongoing before movie release. Indeed, we found that up-trending breeds may have been chosen more often for movies. Our method can be valuable in all studies in which similar confounds may occur. For example, reviewers may prefer to write about particularly good or bad books, rather than about randomly sampled books. Thus reviews may appear to influence sales when, in reality, both may depend on book quality. Hurricane names, on the other hand, are chosen from a predetermined list that is not influenced by first name popularity, and a re-analysis of Westminster Kennel Club Dog Show data using our method confirms that winning breeds do not become more popular. Thus we are not suggesting that previous studies came to incorrect conclusions, but that our method may provide a more accurate estimate of the effect of specific events on popular culture.
